# General aspects, host interaction, and application of *Metarhizium* sp. in arthropod pest and vector control

**DOI:** 10.3389/ffunb.2024.1456964

**Published:** 2024-11-20

**Authors:** Rodrigo Prado Rodrigues de Miranda, Talita Kellen dos Anjos Soares, Daniele Pereira Castro, Fernando Ariel Genta

**Affiliations:** ^1^ Laboratorio de Bioquímica e Fisiologia de Insetos, Instituto Oswaldo Cruz, Fiocruz, Rio de Janeiro, Brazil; ^2^ Instituto Nacional de Ciência e Tecnologia em Entomologia Molecular, Rio de Janeiro, Brazil

**Keywords:** *Metarhizium*, biological control, arthropod, insecticide, vector control

## Abstract

The application of microorganisms as bio-control agents against arthropod populations is a need in many countries, especially in tropical, subtropical, and neotropical endemic areas. Several arthropod species became agricultural pests of paramount economic significance, and many methods have been developed for field and urban applications to prevent their, the most common being the application of chemical insecticides. However, the indiscriminate treatment based upon those substances acted as a selective pressure for upcoming resistant phenotype populations. As alternative tools, microorganisms have been prospected as complementary tools for pest and vectorial control, once they act in a more specific pattern against target organisms than chemicals. They are considered environmentally friendly since they have considerably less off-target effects. Entomopathogenic fungi are organisms capable of exerting pathogenesis in many vector species, thus becoming potential tools for biological management. The entomopathogenic fungi *Metarhizium* sp. have been investigated as a microbiological agent for the control of populations of insects in tropical regions. However, the development of entomopathogenic fungi as control tools depends on physiological studies regarding aspects such as mechanisms of pathogenicity, secreted enzymes, viability, and host-pathogen aspects. The following review briefly narrates current aspects of entomopathogenic fungi, such as physiology, cellular characteristics, host-pathogen interactions, and its previous applications against different insect orders with medical and economic importance. Approaches integrating new isolation, prospection, characterization, delivery strategies, formulations, and molecular and genetic tools will be decisive to elucidate the molecular mechanisms of EPFs and to develop more sustainable alternative pesticides.

## Introduction

*Metarhizium* sp. is a filamentous fungus belonging to the order Hypocreales. It is known for its diverse ecological roles and significant applications in agriculture and biotechnology. This entomopathogenic fungus has garnered considerable attention due to its ability to parasitize a wide range of arthropod hosts, making it a promising candidate for biological pest control strategies (Skinner et al., 2014).

Initially discovered and described by Sorokin in 1883 (Ann et al., 1995), *M. anisopliae* has since been extensively studied for its pathogenic mechanisms and genetic diversity. The fungus exhibits a complex life cycle involving spore germination, insect cuticle penetration and subsequent colonization and growth within the host hemocoel (Bihal et al., 2023). This pathogenicity is facilitated by a range of bioactive compounds and enzymes the fungus produces, which aid in host tissue degradation and nutrient acquisition (Butt et al., 2016).

Beyond its role as a pathogen, *M. anisopliae* has shown potential in various biotechnological applications, including biocontrol of agricultural pests (as it is applied mainly as a mycoinsecticide for certain crops) and vectors of human and animal diseases (Iwanicki et al., 2019; Vivekanandhan et al., 2022; Faria et al., 2023). Its environmental adaptability and ability to thrive in diverse habitats underscore its potential as a sustainable alternative to chemical pesticides (Verma et al., 2023). Furthermore, ongoing research into its genomic structure and molecular biology promises insights into its evolution and adaptation strategies.

In this review, we explore the ecological significance of general fungi, specially Entomopathogenic Fungi (EPFs), with a focus on *Metarhizium anisopliae.* We detail it’s biological characteristics, host-pathogen interaction, and discuss the practical applications of *M. anisopliae*, highlighting its potential contributions to pest management and biotechnological innovations. We aim to provide a comprehensive overview of this intriguing fungus and its impact on sustainable agriculture and environmental management by synthesizing current knowledge and research trends.

The reviewed articles are summarized in **Supplementary Table 1** (see **Supplementary Material**), which includes details on fungus lineage, target arthropod, target insect arthropod order, main results obtained from Metarhizium anisopliae, and bibliographic references.

## General aspects of fungi

Fungi are a taxonomical group possessing profuse biodiversity, containing approximately 2.2 to 3.8 million species (Hawksworth and Lücking, 2017). Fungi are eukaryotic and heterotrophic organisms, single cell (like yeasts) or multicellular macroscopic fungi (like mushrooms). However, they have varied nutritional habits, being able to absorb organic matter from dead organisms (saprophytic) or infect living hosts using specialized propagules of mild duriform or sporiform shape (Mora et al., 2018).

In some reproductive cycles of filamentous fungi (conidia, zoospores, ascospores, basidia, or chlamydospores), specialized structures known as sporophores cleave themselves to form asexual propagules known as conidia with chitinized cell walls. The composition of a conidium wall may include the presence of mannans, galactans, glycans, and chitosan as constituents. After enough nutrient acquisition, the conidium develops in hyphae, which can generate the conidiophores and, sequentially, the sporophores for another cycle (Samson et al., 1988; Blackwell, 2010). In fungal sexual reproduction (found in teleomorphic fungi), the occurrence of plasmogamy and karyogamy of sexual gametes play the main role in genetic diversification and maintenance of the fungal life cycle by the generation of reproductive mycelium. Thereunto, both gametes need sexual complementarity, expressed by the existence of different mating types: mating type +/- or a/α, which varies widely across the taxonomic groups (Zimmermann, 2007; Wallen and Perlin, 2018). It is essential to mention that teleomorphic fungi and anamorphic fungi (fungi that reproduce only asexually) are capable of infecting arthropod hosts (Mora et al., 2018). From this perspective, this review aims to compile the physiological processes of host-pathogen interaction and its historical and applied aspects.

## Entomopathogenic fungi (EPFs)

As previously mentioned, *M. anisopliae* is a highly prospected microorganism for integrated management of pests and vectors. Particularities in its biology are responsible for optimal maintenance of several important features, such as virulence, resistance to abiotic factors (such as desiccation, radiation, and temperature), suppression of other microorganisms with the synthesis of allelopathic molecules, and the ability to evade the host’s immune system once successfully infected (Sevim et al., 2012; Barelli et al., 2016; Donzelli and Krasnoff, 2016; Wang et al., 2016).

Mostly in nature, EPFs are essentially terrestrial beings, capable of infecting their host with the propagation of their conidia via passive horizontal dissemination or auto-disseminating mechanisms, which occurs by direct contact with the corpse of the sporulated host (Juarez et al., 2011; Araújo and Hughes, 2016).

The species status of the *Metarhizium* taxa has undergone phylogenetic reformulations and revisions over time, being promoted to a complex of species and variations, according to Bischoff et al (Bischoff et al., 2009). In this study, the authors redefine the cladistic relationships between species of the genus from quasi-total sequencing of the Genes EF-1α (Elongating Factor), RPB1 (major subunit of RNA polymerase II), β-tubulin and IGS (ribosomal nuclear intergenic spacing region), concurrent with macro and micromorphological aspects, such as size and shape of propagules (classified as conidia, blastospores and “swollen conidia”), size of hyphas, fialidis and conidiophores from 57 isolates.

Derived from this work, the relocation of 6 species was concluded: *M. anisopliae* (formerly a complex permeated by variations), *M. guizhouense*, *M. pingshaense*, *M. acridum*, *M. lepidiotae*, and *M. majus*. Receiving the status of a new species, they promoted: *M. globosum*, *M. robertsii*, and *M. brunneum*, the last being used as synonymy for *M. anisopliae* by several authors.

### Physiological aspects and host-pathogen interaction

#### a) Physiological/molecular changes in pathogen

Fungi interact with different living beings, establishing symbiosis, commensalism, or pathogenicity relationships. Entomopathogenic fungi (EPF) are fungi capable of infecting and developing in arthropod hosts, disposing of a plethora of physical, biochemical, and biological mechanisms (Samson et al., 1988). Although the interactions between fungi and arthropods might be diverse, the classical interface involves adherence of propagules to the host’s cuticle (Butt et al., 2016; Mora et al., 2018). Once in contact with the insect, the conidia (a form derived from the asexual reproduction of the fungus) adheres to the cuticle through physical and biochemical interactions, whose main mechanisms involve hydrophobic interactions promoted by hydrophobins. Such surface proteins are coded in *M. anisopliae* by the HYD1/ssgA and HYD3 genes, producing class I hydrophobins and HYD 2, producing class II hydrophobins (Sevim et al., 2012). The mutation or knockout of these genes affects sporulation capacity, pigmentation, and macromorphological aspects of the fungus, causing a marked decrease in virulence since this impairs the initial mechanism of conidia-cuticle interaction (Sevim et al., 2012; Wang et al., 2016).

Adhesin-like proteins also constitute the machinery of adhesion on the insect´s cuticular surface by *M. anisopliae*. Proteins encoded by MAD I genes (adhesine-like I - able to promote adhesion on the surface of arthropod hosts) and MAD II (adhesine-like 2 - promoter of adhesive interaction on plant surfaces) assist in active adhesion and signaling for the budding of the appressorium, followed by penetration into the host organism and further colonization. In the case of hydrophobins, once the propagule adheres, the protein is degraded and removed from the propagule wall (Wösten, 2001; Wang and St. Leger, 2007; Wyrebek and Bidochka, 2013; Yang et al., 2023).

According to Wang and Leger (Wang and St. Leger, 2007), the imbalance in MAD I expression promoted delays in germination, low differentiation in blastospores, and high reduction in virulence to *Manduca sexta* caterpillars. At the same time, the impairment of Mad II expression did not compromise pathogenicity in the animal host (Wang and St. Leger, 2007).

Similar to adherence, recognition is essential for the infective capacity of the fungus. The enzyme glyceraldehyde-3-phosphate dehydrogenase is another wall constituent of *M. anisopliae* that is also responsible for molecular adhesion mechanisms. This enzyme acts as an adhesine-like protein. It is differentially synthesized after exposure to different carbon sources, also composing the enzymatic machinery that will promote the lysis of the host cuticle (Broetto et al., 2010). After recognition and adhesion, cascades of biochemical signs, such as those of protein class kinases A, promote a change in the composition of the fungal cell wall. This change allows the increase of turgor, favoring the budding and emergence of the appressorium (Fang et al., 2009; Butt et al., 2016).

During differentiation, the fungus utilizes energy reserves stocked up as carbon sources like lipids, trehalose, glycogen, and erythritol (Hallsworth and Magan, 1996). Perilipin-like proteins are evolutionarily conserved in various organisms, such as fungi, frogs, and mammals, and are responsible for triacylglycerol breakdown (Miura et al., 2002; Bickel et al., 2010). In the genus *Metarhizium*, perilipin-like proteins are encoded by the Mpl 1 gene. They can convert these sources of carbon from lipid droplets, which will be consumed mainly during the differentiation of fungal apressorium at the time of penetration into the host cuticle (Wang and St. Leger, 2006; Bickel et al., 2010; Butt et al., 2016).

The key enzymes that promote penetration of the appressorium are chitinases and proteases (Butt et al., 2016). The Pr1 serine endopeptidases are the main proteases in *Metarhzium* sp and consist of 11 isoforms distributed throughout the genus, subdivided into two classes and three subfamilies (Andreis et al., 2019). In synergism, such different isoforms of Protease Pr1 together with lipases, N-acetylglycosaminidases, and chitinases, will aid in cuticular degradation, allowing the invasion of the arthropod hemocele and consequently trigger immunological responses (Small and Bidochka, 2005).

In the context of later stages of infection, EPF propagules developed adaptations to avoid the humoral and cellular components of the host, as they stimulate the host’s immune system. According to Verma et al (Wang and St. Leger, 2006). one of the most notorious examples of avoidance mechanisms is performed by the MCL1 gene in *M. anisopliae*. This gene encodes a hydrophilic trimeric protein containing an N-terminal region with 14 cysteine residues, negatively charged with tandem regions, and a C-terminal region. The C-terminal contains an attachment site to the cell wall, dependent on glycosylphosphatidylinositol, which, simultaneously with other physiological aspects, provides the fungus with anti-adhesive capacity, making it difficult to adhere to plasmatocytes and other phagocytic hemocytes.

Along with the immunological pressures exerted on the propagules of EPFs, the fungus needs adaptation to the osmotic pressures of the host’s hemolymph. In *M. anisopliae*, the Mos1 gene encodes the transmembrane protein Mos1, structurally similar to the osmotic regulators found in yeasts such as *Candida albicans* and *Saccharomyces cerevisae*, the specific SHO1 and SLN1 receptors, that are positive regulators of the map kinase pathway controlling the cell cycle (Alonso Monge et al., 2006; Tatebayashi et al., 2007). Widely distributed in Fungi, these osmoregulatory proteinscan in *M. anisopliae* can preserve the integrity of the cell wall when exposed to oxidative stress. It was observed that its silencing reduced the viability of the fungus in the hemocoel, further compromising the regulation of genes related to growth factors and differentiation in the hemolymph during host colonization (Wang et al., 2008).

Furthermore, in yeasts of the species *Saccharomyces cerevisae*, PacC/Rim101 transcription factors regulate gene expression in an alkaline environment, repressing it when in a very acidic one (Peñalva et al., 2008). However, in *Metarhizium robertsii*, another generalist EPF, this same gene family is essential for evading the immunological components of the host; besides being responsible for survival in an osmotically stressful environment, penetration of the cuticle, colonization of hemolymph in the host, and other aspects that characterize virulence (Huang et al., 2015; Xie et al., 2019).

Other fungal molecular and cellular mechanisms for evading the host immune system remain unknown and require further studies since such interaction interfaces present complexities not only at physiological but also at the evolutionary-adaptive level. After overcoming the physiological barriers of the insect, conidia differentiate into blastospores, which propagate inside the hemocoel. After successful colonization of the host, the fungus emerges by lytic activity mediated by proteases, chitinases, lipases, acetylglycosaminidases, and secondary metabolites of the most varied molecular classes, breaking the cuticle from the inside to assume the filamentous form in the exterior of the host carcass. Then, sporulation and dispersion of propagules take place (Barelli et al., 2016; Butt et al., 2016).

#### b) Molecular level perspective in the host after host-pathogen interaction

**Figure 1:**
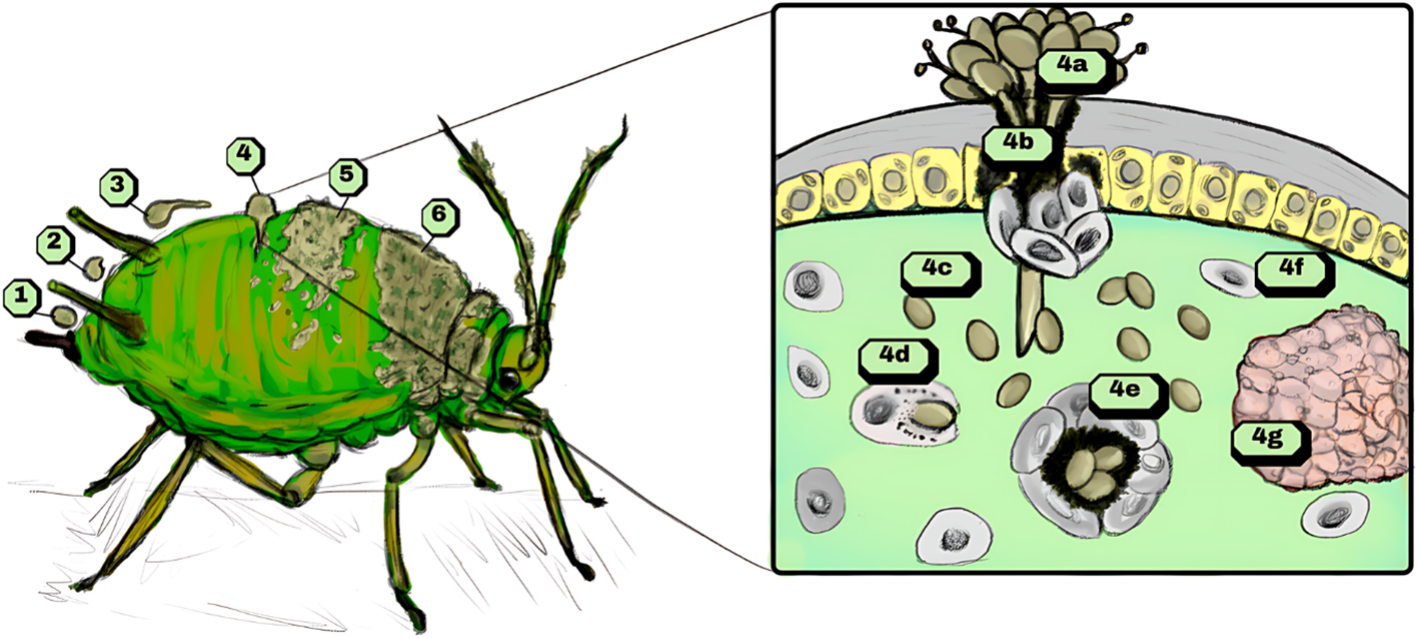
Process of infection of entomopathogenic fungi *Metarhizium* sp. in an insect host. Recognition and adherence are depicted in infection phases 1 and 2. Appressorium formation is stimulated shortly after adherence (3), and enzymatic secretion along with penetration force results in the invasion of fungi in the host celome (4). The host’s immune reaction resulting from the infection may manifest by humoral and cellular responses, as follows: melanization from phenoloxidase cascade (4a), which surrounds and encapsulates the fungal propagules with circulating cascade precursors. Simultaneously, cellular encapsulation mitigates the infection by recruiting other immune cells (4b). As fungi differentiate from hyphal structures to blastospores in the celome (4c), phagocytosis by plasmatocytes and granular cells (4d), deposition of melanin by oenocytes (4e) and formation of nodules around propagules by recruited immune cells (4f, 4g) are common immunological responses to fungal infection. After evasion from the host’s immune system, the pathogen spreads (5) and finishes its cycle, producing spores across the cuticle (6).

After the invasion of the insect’s celomatic cavity, the immune system is activated through recognition by receptors associated with molecular patterns (Lu et al., 2014) to counteract the presence of a pathogenic foreign microorganism. This invasion is illustrated in **Figure 1**, which depicts the different stages of *Metarhizium* sp. infection (labeled 1 to 6) in an aphid, along with the detailed immune system response of the insect at stage 4.

During the infection, one of the first components of the host’s humoral immune response to be activated is the phenoloxidase (PO) cascade. The PO cascade consists of the sequential activation of several serine proteases, which will cleave the prophenoloxidase zymogen (pre-proPO) into its second form, prophenoloxidase (proPO). Other serine proteases of unknown nature cleave the second zymogen for the activation of proPO into PO. Then, the active PO catalyzes the oxidation of phenols in quinones, which are components of the polymerization of melanin, a molecule that will control the propagation of the pathogen (Cerenius and Söderhäll, 2004; Binggeli et al., 2014; Lu et al., 2014).

As a result of the recognition of molecular patterns associated with pathogenic microorganisms, the synthesis of antimicrobial peptides (AMP) is essential to combat a variety of infections. These AMPs are small molecules with, on average, less than 10 kDa (equivalent to peptides between 12 and 50 amino acids). The fat body is the main site of expression and synthesis of these peptides. This organ comes ontologically from the mesoderm and is the host organism’s largest source of immunological responses (cellular and systemic) (Lemaitre and Hoffmann, 2007). About 20 immunologically induced AMPs have been described, with great antimicrobial specificity, depending on the invasive pathogen. For example, attacins have a more significant effect on gram-negative bacteria, defensins on gram-positive bacteria, and drosomycins and cecropins have higher antifungal activity (Lemaitre and Hoffmann, 2007; Cohen et al., 2020).

Most of these AMPs occur throughout the Hexapoda class and can be divided into three groups: (i) peptides rich in proline and glycine residues, (ii) defensins with three to four disulfide bridges between conserved cysteine residues, and (iii) cecropins, linear peptides that are rich in alpha-helix (Wu et al., 2018).

As mentioned above, drosomycins and cecropins, among other antimicrobial peptides, have antifungal capabilities for controlling a possible EPF infection. The signaling cascade responsible for this particular response is the Toll pathway, which is highly conserved in mammals. In most insects, this pathway encompasses recognizing specific fungal molecules such as chitosan and beta-1,3-glucans, for example (De Lima Batista et al., 2018).

After recognizing such fungal wall components, the Toll pathway is activated by the binding of one of the cleaved forms of the Spätzle cytokine, which is still immature. After successive cleavages of the cytokine, it binds as a dimer in the Toll receptor, promoting intracellular recruitment of three proteins containing Death domains (molecular subunits that constitute adapters in receptors responsible for the transfer of chemical groups (kinases in *Drosophila* and other Diptera, for example) or, in mammals, by activation of the procaspase pathways 8 and 10 (Kaufmann and Hengartner, 2001; Cooper et al., 2009; Walderdorff et al., 2019).

Proteins as MyD88, Tube, and Pelle will act in the negative regulation of other systemic immunological pathways (more precisely, IMD and Cactus) by the ubiquitination of immunological components that are posteriorly directed to the proteasome. In the cells of the fat body, the transcription factors positively regulated (Dif and Dorsal) by the action of these three proteins act on specific genes of cecropins, drosocins, metchnikowins, and other AMP genes (Ligoxygakis et al., 2002; Tauszig-Delamasure et al., 2002; Lemaitre and Hoffmann, 2007).

Another immune response against EPF infections in insects is the cellular response, related to hemocytes activated after recognition of Pathogen Associated Molecular Patterns (PAMPs). There are different types of hemocytes in the insect hemolymph, each with a specific activity. Prohemocytes are pluripotent immature cellular populations, capable of differentiating mainly in lamelocytes (these occurring mostly in larvae of holometabolous individuals), granulocytes, and plasmatocytes (Pondeville et al., 2020). Plasmatocytes in different insect species are capable of phagocyte invading microorganisms (Ratcliffe and Gagen, 1977; Lackie, 1988; Walderdorff et al., 2019). Granulocytes, in synergism with plasmatocytes, can encapsulate and promote the nodulation of foreign agents, whether cells or parasitic/parasitoid organisms. Oenocytoids and crystal cells play an essential role in the PO cascade. The inactive PPO is stored in these cells, and after recognition of PAMPs, they degranulate, releasing the enzyme into the hemolymph (Lu et al., 2014). The hemocyte types vary according to the insect species; some cells such as spherocytes and coagulocytes have unknown functions. However, they may play physiological roles related to the cellular and humoral immune systems (Lemaitre and Hoffmann, 2007; Hernandez et al., 2010; Cho and Cho, 2019; Ross et al., 2020).

## Historical aspects of arthropods’ control

Taking together the environmental benefits, mechanisms of action, and practical aspects of EPF biopesticides, several studies have been carried out on *M. anisoplae* and its activity in different orders of Insecta. These studies aim to prospect the potential for large-scale employment against the most diverse pests and vectors. In this context, it is relevant to consider the most important facts related to insecticide evolution for arthropod pests and vectors and when the EPF’s biopesticides started to be developed.

Along with the ascension of civilization and agriculture populations of insect pests and vectors gradually began to adapt to human environments in a coevolutionary process. This led to the prospection of measures to restrict these organisms’ population size, aiming at reducing their impact on health and economy. As the most well-known measures used in the current scenario, chemical insecticides are broadly utilized as populational deterrents. According to the World Health Organization (WHO), the most common chemical insecticides are currently represented by molecular classes such as pyrethroids, organochlorides, and organophosphates, which act in the target organisms as neurological disruptors (Nicolopoulou-Stamati et al., 2016; Wilson et al., 2020).

The modern advent of chemical pesticides as a major population control measure for pests and vectors began in the 1940s, using the insecticide dichlorodiphenyltrichloroethane (DDT) to control *Anopheles* mosquitoes that transmit the etiologic agent of malaria, the *Plasmodium* parasite. Other chemical classes gradually replaced this insecticide due to its high toxicity and residuality. New active substances against insects were discovered with several modes of action and properties, such as acetylcholinesterase (AChE) inhibitors, gaba-gated chlorine channel blockers, sodium channel modulators, and nicotinic acetylcholine receptor (nAChR) competitive modulators. However, all these compounds target the same insect’s physiological system, the nervous, providing fast action (Casida and Quistad, 1998; Bate, 2007; Oberemok et al., 2015; Nicolopoulou-Stamati et al., 2016; Isman, 2020; IRAC, 2023). Although they have been and necessary for insect control, these chemical compounds have triggered detrimental effects, similar to DDT, such as poisoning of non-target beneficial species as well as mammals. However, current chemical classes, such as pyrethroids, present low toxicity to humans. In addition, the residual accumulation of these substances in various trophic chains poses a significant threat to ecosystems. Besides that, inappropriate use of chemical insecticides may lead to the selection of resistant populations with physiological changes for the individual, such as increased cuticle thickness, increased synthesis of detoxifying enzymes (e.g., cytochrome p450 class proteins), behavioral changes culminating in the avoidance of the control measures, and other adaptations (Hemingway and Ranson, 2000; Puinean et al., 2010; Kasai et al., 2014; Nicolopoulou-Stamati et al., 2016; Zalucki and Furlong, 2017).

The investigation of alternative ways to contour the obstacles linked to the use of chemical pesticides led to the prospection of new classes of products, for example, plant-based insecticides, which can act as hormonal analogs or antagonists, such as neem. Also, microorganism-based pesticides can infect the insects, leading to their death. It exerts negative ecological pressure on target organisms, being more selective and causing less residual and harmful effects to the environment, being more sustainable to pests/vectors populational management (Carvalho, 2017; Senthil-Nathan, 2020).

The plant-based insecticides (derived from secondary plant metabolites or phytochemicals) are highly prospected products for pest/vector management, acting by different mechanisms over its physiological system, whether in the cellular, tissue, or systemic levels. For example, bruchelin and podofilotoxin, two molecules belonging to the class of neolignans, were able to alter the excretion system of the blood-feeding triatomine *Rhodnius prolixus* (Hemiptera, Reduviidae), an important vector in the transmission of *Trypanosoma cruzi*, the etiological agent of Chagas disease. These compounds reduce diuretic hormones in the hemolymph and Malpighian tubules (Garcia et al., 2000). It was also seen that gonadulin, an insulin-like peptide, is highly expressed in the calyx of the *R. prolixus* females’ reproductive system. This peptide is associated with the modulation of ovulation and oviposition, implying that the insulin cascade is essential for egg production and is, therefore, a possible target for populational control of this insect (Leyria et al., 2023).

In *Aedes aegypti* (Diptera, Culicidae, a mosquito of great sanitary relevance for its’ competence as a vector of several viral diseases like yellow fever, dengue, Zika, Mayaro, and chikungunya), phytochemicals have a broad spectrum with systemic action. Such substances are effective in promoting changes in the cuticle (tissue level) and membrane components (such as the lipid bilayer), different proteins, such as enzymes, transmembrane receptors, cellular messengers, and transmembrane ionic channels, and may also affect the insect at the genetic level (DNA and RNA) (Senthil-Nathan, 2020). For example, Workman et al. (2020) observed the larvicidal potential of orange essential oil encapsulated in the *Saccharomyces cerevisae* yeast. The compound is formed mainly by limonene (89.6%), having as secondary constituents myrcene (2.4%), γ-terpinene (1.6%), and 8.2% of other molecules, causing lethality in 90% of larvae treated at the concentration of 18.9 mg/L (or 18.9 ppm). Besides that, the encapsulation process promoted the dispersion in the water, as well as the ingestion of the product by the insects, optimizing its insecticide effect (Workman et al., 2020).

Biopesticides from microorganisms or their metabolites are used to control different species of arthropods with medical and economic relevance, being considered, as well as phytochemicals, substitutes for conventional chemical insecticides. The formulation and constitution of biological pesticides can vary greatly, from virulent molecules isolated from fungi, active in the interior of the target, to infective microorganisms, predators, or parasitic/parasitoid macroorganisms, in addition to possible combinations between both (Chandler et al., 2011; Balog et al., 2017). For example, in the case of the bacteria *Bacillus thuringiensis* var. *israelensis* (Bti) and *Lysinibacillus sphaericus*, both the microorganism itself and its insecticidal crystalline inclusions (ICPs) can be marketed for field and semi-field application, with its formulations focused both on aquatic environments or on terrestrial environments with varying moisture rates (FAO and WHO, 2017; Thakur et al., 2020).

Belonging to the order Rickettsiales, the endosymbiont gram-positive α-proteobacteria of the genus *Wolbachia* likewise represents a promising perspective for the control of various vectors, including *Ae. aegypti*. From a specific mechanism in the symbiont-host interaction known as cytoplasmic incompatibility, mosquitoes may be led to the induction of reproductive alterations such as parthenogenesis and feminization, which is acquired from the crossing of transfected males (infected with bacterial strains like wMel) with wild females. The mechanism may further result in a constituent offspring of unviable eggs, infertile individuals, or insects refractory to viral infections (Stouthamer et al., 1999; Caragata et al., 2019; Reyes et al., 2021; Tantowijoyo et al., 2022). The programmed releases of *Ae. aegypti* males transfected with the bacterium in endemic and high-risk areas is being carried out around the globe, with the objective of gradual replacement of the populations of the vector that are competent for the development of arboviruses by modified refractory populations (Ross et al., 2020).

Similarly, EPFs constitute a group of microorganisms studied for the development of sustainable insecticides for the control of arthropods. The prospection of the insecticide capacity of EPFs began with the Russian microbiologist Elie Metchnikoff, who first described *M. anisopliae* in 1878 and was the father of cellular immunology, being responsible for pointing out, in 1883, the occurrence of macrophages involved in the immune system of anurans (Ann et al., 1995).

In 1878, after the discovery of the infection that affected pest beetles of the Austrian genus *Anisoplia* by a fungus, the pathogen was baptized by this scientist as green muscaridine, with the binomial nomenclature *Entomopthora anisopliae*. Later, in 1880, this fungus was renamed *Isaria destructo*r, and finally, in 1883, by Sorokin, definitively defined as *M. anisopliae* (Ann et al., 1995; Merien, 2016).

EPFs are present in various orders and clades of organisms (COOKE et al., 1892). There are four groups of parasitic fungi with activity against insects: Phylum Zoopagomycota - Order Entomophtorales, Phylum Mucormycota – Order Mucorales Phylum Oomycota – Orders Saprolegniales and Lagenidiales, and Phylum Ascomycota (Samson et al., 1988; Spatafora et al., 2016). More recent studies describe the occurrence of these microorganisms in 6 different phyla (including Basidiomycota and the removed phylum Deuteromycota) and 12 classes (Abdelghany, 2015). They are characterized by their great complexity in the life cycle and a high degree of infectivity over general or specific hosts. The following topic will revise the advances on controlling main groups of arthropods that are relevant in the economic and sanitary sphere using *Metarhizium* spp.

## Application of EPFs against insect orders

Microbial pesticides currently consist of about 1.3% of the total global pesticides used to control economically relevant insects (Um et al., 2018). Inserted as its constituents, EPF-based pesticides are present in 90% of the applications of new biopesticide formulations (Hajek and St Leger, 1994). The group has between 700 and 1000 species already described (St. Leger and Wang, 2010; Mora et al., 2018). The most studied and used EPF in the development of formulations for biopesticides are *Beauveria bassiana* (Balls) Vuill (33.9%), *Lecanicilium* sp. (6.4%), and *M. anisopliae* (Metch) *sensu lato* (33.9%) (Faria and Wraight, 2007). The main orders of arthropods targets of bioinsecticides are Lepidoptera, Coleoptera, Diptera, Hemiptera, and Ixodida. Below, we present a review of the fungal bioinsecticides used to control each main order of arthropods.

**Lepidoptera:** In the last fifteen years, studies focused on pest control of species of the order Lepidoptera by *M. anisopliae* involved Gelechiidae and Noctuiidae.

Lepidopteran insect management consists of a solid state of art in current biological control literature, since a diversity of economically important plants are highly affected. Some of the latest advances in the prospection and utilization of *Metarhizum* spp. Against Lepidopteran are summarized in **Table 1**.

**Table 1:** Summary of the studies on the application *Metarhizum* sp. for controlling pests and vectors of different orders of arthropods.

Order Lepidoptera					
Family	Species	Economic Relevance	EPFs tested	Effect	Ref.
Gelechiidae	*Tuta absoluta*	Tomato pest	*Metarhizium anisopliae* (ESALQ9, PL43, PI47, URPE6 URPE19)*, Beauveria bassiana* (ESALQ447, ESALQ900, CG001, CPATC053, CPATC057) plus insecticides chlorfenapyr, spinosad, indoxacarb, abamectin, and neem	*M. ansipliae* URPE6 and URPE19 were more pathogenic to eggs and first instar larvae. Furthermore, the first mentioned strain was compatible to an average concentration of chlorfenapyr, while the last were compatible with abamectin for optimized application.	(Pires et al., 2010)
	*Phthorimaea operculella*	Potato pest	*M. anispliae* (unidentified strain)	Suspensions at concentrations of 10^7^ to 10^3^ were formulated for mortality tests. The results were heterogeneous, with LCs50 ranging between concentrations of 10^5^, 106 and 10^7^ conidia/mL, resulting in mortality rates ranging from 21.2% to 52.3%.	(Pandey et al., 2015)
Noctuiidae	*Spodoptera frugiperda*	Cotton, soy, corn	*M. anisopliae/B. bassiana* plus chlorpyriphos/spinosad	High mortality	(El-Katatny, 2010; Rivero-Borja et al., 2018; Han et al., 2023)
	*Spodoptera littoralis*	Cotton, avocado, pea beans, sugar cane	*M. brunneum* ORP-27, ORP-13, and ORM-40	Mortalities of 49,79%, 58,78%, and 46% after 13 days post-infection, lethal concentrations of 1.68× 10^7^, 2.10× 10^7^, and 2.25× 10^7^ propagules/mL.	(Şahin and Yanar, 2021)
	*Alabama argillaceae*	Cotton worm	*M. anisoplae* and *B. baussiana* with predator bedbug	EPF extinguished the predator bedbug and had no synergistic effects for such circumstances.	(França et al., 2006)
Plutellidae	*P. xylostela*	Cabbage, broccoli and other cruciferous plants	Metarhizum brunneum ESALQ E9, IPA-207, ESALQ 860, IPA-204, UFPE 3027	High lethality for larvae, 58-96%, 10^5^-10^8^ conidia/mL.	(Silva et al., 2003; Hernandez et al., 2010; Callejas-Negrete et al., 2015; Shakeel et al., 2018; Cervantes Quintero et al., 2020)
Tortricidae	*Thaumatotibia leucotreta*	Orange, macadamia,cotton pest	*M. brunneum* (ICIPE 30, ICIPE 18, ICIPE 78, ICIPE 62, ICIPE 69, ICIPE 63, ICIPE 20, ICIPE 7, ICIPE 74, ICIPE 656, ICIPE 68, ICIPE 40, ICIPE 315, ICIPE 31, ICIPE 22, ICIPE 725, ICIPE 676), and *B. bassiana* (ICIPE 720, ICIPE 283, ICIPE 273, ICIPE 279, ICIPE 647)	12 strains with mortality ranging from 58.8 to 94,2% and horizontal transmission from sporulating corpses.	(Mkiga et al., 2020)
Lyonetiidae	*Leucoptera coffeella*	Coffee pest	*M. brunneum* (RD-20.120) and *M. robertsii* (RD-20.114)	Lethal and feeding inhibition action.	(Jaber and Ownley, 2018; Franzin et al., 2022)

Order Coleoptera					
Family	Species	Economic Relevance	EPFs tested	Effect	Ref.
Curculionidae	*Listronotus maculicollis*	Poaceae pest	*M. anisopliae*	Mortalities 67-89% and 85-100% (5.2 and 7.3x10^9^ granules/g). Inefficient in semi-field conditions.	(Koppenhöfer et al., 2020)
	*Hylobius abietis*	Conifers pest	*M. brunneum*, *B. bassiana*, *B. caledonica*, and *Candida albicans*	Modulation of insect immunity (PO, glycosidases, antimicrobial peptides)	(Ansari and Butt, 2012; Namara et al., 2018)
	*Rhynchophorus ferrugineus*	Palm beetle	*Metarhizium* sp. ZJ-1	Mortality 60% and 100% (10^6^ and 10^8^ propagules/mL) after 10 days. 50% Lethal time of 1.66 days (10^8^ propagules/mL)	(Sun et al., 2016)
	*Anthonomus grandis*	Cotton pest	*M. anisopliae*, 28 different strains	High integrated lethality with Recommended Field Application Ranges (RFAR) 1.2 x 10^7^ conidia/mL (RFAR 20) and 1.13 x 10^7^ conidia/mL (RFAR 50)	(Nussenbaum and Lecuona, 2012)
Chrysomelidae	*Cerotoma arcuata*	Legumes pest	*B. bassiana*, *M. anisopliae* (multiple strains) and *Bacillus thuringiensis*	Treatment with conidial suspension (10^8^ conidia/mL) of *M. anisopliae* CG 210 and CG 321 caused mortality ranging from 80 to 100% within7 days, while *B. bassiana* strain CG 156 e CG 213 caused 100% mortality in the period.	(Teixeira and Franco, 2007)
Brentidae	*Cylas formicarius*	Sweet potato	*M. anisopliae* strain QS155 and QS002-3	High virulence strain QS155 caused over 80% repellence to male adults and over 70% repellence to female adults in comparison to Low virulence strain QS002-3, which caused 29.3% repellence.	(Dotaona et al., 2017)
Cerambicidae	*Anoplophora glabripennis*	Rose bushes, apple trees, mulberry	*M. brunneum*	High mortality between 22 and 24 days after treatment (10^8^ conidia).	(Clifton et al., 2020)
Elateridae	*Limonius californicus*	Sugar beet, potato	*M. brunneum*	High mortality dose dependent.	(Ensafi et al., 2018)
Scarabeidae	*Popillia japonica*	Grapes, corn, peas, peaches, plum	*M. brunneum*	Pure granules LC50 equivalent to 1,9 x 10^7^ was observed for *P. japonica* and granules plus microesclerotia LC50 equivalent of 5,9 x 10^7^ was detected for *P. japonica*	(Behle and Goett, 2016)
	*Phyllophaga* sp	Soy, wheat, coffee	*M. brunneum*	Pure granules LC50 equivalent to 7,1 x 10^6^ was observed for *Phyllophaga* and granules + microesclerotia LC50 equivalent of 5,1 x 10^7^ was detected for *Phyllophaga*	(Behle and Goett, 2016)
Curculionidae	*Hypothenemus hampei*	Coffee	*Metarhizium* sp. MMR-M1	No significant mortality (10^5^, 10^7^, and 10^9^ conidia/mL).	(Chuquibala-Checan et al., 2023)

Order Diptera					
Family	Species	Economic Relevance	EPFs tested	Effect	Ref.
Culicidae	*Aedes aegypti*	Arbovirus vector	*M. brunneum* strain ARSEFF 4556	Mechanism of virulence through feeding and apoptosis of intestinal cells.	(Butt et al., 2013)
	*Aedes aegypti*	Arbovirus vector	*M. anisopliae* strain ESALQ818	Synergism with Neem oil.	(Gomes et al., 2015)
	*Aedes aegypti*	Arbovirus vector	*M. anisopliae* strain IP46	Action of propagules with vegetable or mineral oil, and diatomaceous earth.	(Rodrigues et al., 2019)
	*Aedes aegypti*	Arbovirus vector	*M. anisopliae/brunneum* strains ARSEF V275 and 4556	Synergism with Phenyl thiourea.	(Prado et al., 2020)
	*Aedes aegypti*	Arbovirus vector	*M. anisopliae*	Contamination by contact with tissues impregnated with conidia.	(Paula et al., 2013)
	*Aedes aegypti*	Arbovirus vector	*M. anisopliae* strains ARSEF V275, 4556 and 3297	*Aedes aegypti* larvae were more tolerant to the three strains of *M. anisopliae* in both formulations (wet and dry conidia)	(Greenfield et al., 2015)
	*Culex quinquefasciatus*	Filariasis and Arbovirus vector	*M. anisopliae* 3 strains ARSEF V275, 4556 and 3297	Strain ARSEF 4556 was more virulent in comparison to the other two strains, with LT (lethal time) 50 ranging from 0.3 to 1.1 days. *Culex quinquefasciatus* and *Anopheles staphensi* were more susceptible than *Aedes aegypti* to this strain. No significant difference was observed between formulation (dry or aquous/wet).	(Greenfield et al., 2015)
	*Anopheles stephensi*	Malaria vector	*M. anisopliae* 3 strains ARSEF V275, 4556 and 3297	Strain ARSEF 4556 was more virulent in comparison to the other two strains, with LT (lethal time) 50 ranging from 0.3 to 1.1 days. *Culex quinquefasciatus* and *Anopheles staphensi* were more susceptible than *Aedes aegypti* to this strain.	(Greenfield et al., 2015)
	*Culex quinquefasciatus*	Filariasis and Arbovirus vector	*M. anisopliae* and *B. bassiana*	Synthetic attractants in dry formulations of EPF conidia.	(Paula et al., 2018)
	*Aedes albopictus*	Arbovirus vector	*M. anisopliae* and *B. bassiana*	Synthetic attractants in dry formulations of EPF conidia.	(Paula et al., 2018)
	*Anopheles* sp.	Malaria vector	*M. anisopliae* and *B. bassiana*	Synthetic attractants in dry formulations of EPF conidia.	(Paula et al., 2018)
	*Culex quinquefasciatus*	Filariasis and Arbovirus vector	*M. anisopliae*	Virulence and effect on enzymatic activities of chlorpyrifos-resistant strain.	(Ismail et al., 2020)
	*Aedes albopictus*	Arbovirus vector	*M. anisopliae*	Transgenerational effects and populational control.	(Shoukat et al., 2019)
	*Anopheles gambiae*	Malaria vector	*M. anisopliae*	Effects of diet and age on treatment outcome.	(Mnyone et al., 2011)
	*Anopheles stephensi*	Malaria vector	*M. anisopliae* Ma4 and Ma-NBAIR, and *B. bassiana* Bb5a and Bb-NBAIR	Significant adult mortality (72.5 and 88.75% for Bb5a and BbNBAIR; 57.5% and 48.75% for Ma4 and Ma-NBAIR) after 10 days of exposition to cement or mud panels with 10^7^ propagules/mL. Higher larval mortality for *B. baussiana* than *M. anisopliae*. Ma4 delay pupation to 11 days vs 6 days in the control.	(Renuka et al., 2023)
Muscidae	*Musca domestica*	Mechanical vector (several pathogens)	*M. anisopliae* with cypermethrin and chlorpyrifos	Administration of sublethal doses of ChCy with a certain concentration of conidia (10^6^ propagules/mL) caused a mortality ranging from 62 to 72% in 5 days.	(Ong et al., 2017)
Tephritidae	*Ceratitis capitata*	Fruit fly	*Metarhizium brunneum* plus radiation	*M. brunneum* strain EAMa 01/58-Su showed tolerance to UV-B and was used for testing on insects. *C. capitata* adults were treated with 5 suspensions (10^4^ - 10^8^ conidia/mL) of conidia and irradiated with UV-B to assess virulence, viability and germination. It was observed that such exposure to UV-B did not significantly interfere with such parameters. The time of exposure to UV-B directly interfered with the mortality exerted by the fungus on the insect, where 6 hours of exposure resulted in 56.7% mortality, 24 hours in 43.3%, and 48 hours resulted in 30% mortality.	(Fernández-Bravo et al., 2017)
	*Zeudogacus cucurbitae*	Cucurbitaceae plants, melon, watermelon	*M. anisopliae* Ma31, MaD, and *B. bassiana* Bb13, Bb14, Bb337, Bb338, Bb339, and Bb353	Larval mortality from 1.49-6.33% (MaD) to 5.82-21.70% (Bb337), and from 18.6% (MaD) to 61,1% (Bb337) after 10 days post-treatment. Pupal mortality over 50% (10^5^-10^10^ propagules/mL). High sporulation rates from insect cadavers (Bb337, Bb338).	(Hintènou et al., 2023)
Ceratopogonidae	*Culicoides* sp.	Cattle bluetongue virus vector	*M. anisopliae*	Laboratory and simulated field conditions: strains, doses, population size effects.	(Ansari et al., 2010; Nicholas and Mccorkell, 2014; Ansari et al., 2019; Cazorla and Campos, 2020)
Psychodidae	*Phlebotomus duboscqui*, *P. papatasi*	Leishmaniasis vectors	*M. anisopliae*	Effects of population density and number of generations	(Ngumbi et al., 2011; El-Shazly et al., 2012; Zayed et al., 2013)

Order Hemiptera					
Family	Species	Economic Relevance	EPFs tested	Effect	Ref.
Pentatomidae	*Podisus nigrispinus*	Predatory bedbug	*M. anisopliae* and *B. bassiana*	EPFs extinguished the predator	(França et al., 2006)
Delphacidae	*Peregrinus maidis*	Corn viruses’ vector	*M. anisopliae* and *B. bassiana*	Colonization of hemocele and sporulative cycle in 6 days	(Toledo et al., 2010)
Pyrrochoridae	*Dysdercus peruvianus*	Cotton pest	*M.anisopliae*	Role of host’s ectophosphatase in fungal pathogeneicty	(Cosentino-Gomes et al., 2013)
Diaspididae	*Aulacaspis tubercularis*	Avocado and papaya pest	*M. anisopliae* and *B. bassiana*	Efficacy of formulations of 3 strains	(Sayed and Dunlap, 2019)
Monophlebidae	*Icerya seychellarum*	Broad spectrum plant pest	*M. anisopliae* and *B. bassiana*	Efficacy of formulations of 3 strains	(Sayed and Dunlap, 2019)
Aphidae	*Aphis glossypii*	Polyphagous plant pest	*M. anisopliae* with flonicamid, imidacloprid, nitenpyram, dinotefuran, pymetrozine, pyriproxyfen, spirotetramat or matrine	Mortalities from 17.08 (fungi alone) to 91.68% (EPF plus flonicamid)	(Carletto et al., 2009; Nawaz et al., 2022)
Reduviidae	*Triatoma infestans*	Chagas disease vector	*M. anisopliae*, *M. robertsii*, *M. flavoviridae*, and *Isaria* sp.	Higher mortality rates with Metarhizium than with Isaria	(Rocha and Luz, 2011)
	*Triatoma infestans*	Chagas disease vector	*B. bassiana*, *M. anisopliae*, *Gliocladium virens*, and *Talaromyces flavus*	High efficacy for *B. baussiana* and *G. virens* against Mexican insect populations	(Vazquez-Martinez et al., 2014)
	*Rhodnius prolixus*	Chagas disease vector	*M. anisopliae* and *B. bassiana* plus *Trypanosoma cruzi*	Colonization resistance between pathogens	(Rv et al., 2014; Garcia et al., 2016)
Cimicidae	*Cimex lectularius*	Mechanical vector of several pathogens	*M. anisopliae*	Effects of moisture, topical or oral treatment	(Ulrich et al., 2014)

Order Ixodida					
Family	Species	Economic Relevance	EPFs tested	Effect	Ref.
Ixodidae	*Riphicephalus microplus*	Cattle tick	*M. anisopliae* with oil formulations	Efficient dispersion with the viability of propagules	(Polar et al., 2005)
	*Rhipicephalus sanguineus*	Brown dog tick	*M. anisopliae* and *B. bassiana* emulsified with oil and cellulose gel	Higher mortality with fungal viability	(Reis et al., 2008)
	*Ixodes scapularis*	Deer tick, Lyme disease vector	*M. anisopliae* with permethrin	LD_50_ of 10^7^ (laboratory) or 10^9^ (field).No synergy between treatments	(Hornbostel et al., 2005)
	*Riphicephalus microplus*	Cattle tick	*M. anisopliae* plus deltamethrin	Low mortalities in treated field populations. Low egg production after treatment	(Bahiense et al., 2008)
	*Riphicephalus microplus*	Cattle tick	*M. anisopliae*	Two subtilisinic protease inhibitors BmSI-6 and BmSI-7 have no effects on the interaction with the EPF	(Sasaki et al., 2008)
	*Ixodes scapularis*	Deer tick, Lyme disease vector	*B. baussiana* and *M. anisopliae* with bifentrin	Higher efficacy with *M. anisopliae* than with *B. baussiana*. Higher mortality in the bifentrin controls	(Stafford and Allan, 2010)
	*R. microplus*	Cattle tick	*M. anisopliae*	Protection of fungi with aqueous or oily formulations against abiotic factors. Integrated management with low doses of chemical insecticides plus EPFs	(Beys-da-Silva et al., 2020)

**Coleoptera:** Studies on the use of *M. anisopliae* (and other EPFs) in laboratory and semi-field conditions, with integrations to other insecticides or not, against the order Coleoptera, occur mainly using the family Curculionidae as a target

Alternatively, other Coleoptera representatives with the most varied economic relevance are aimed for populational management, such as the families Chrysomelidae Brentidae, Cerambicidae, Elateridae, Scarabeidae, among others, already have a history of investigations for single and integrated management with EPFs in the most varied formulations (see **Table 1** for details).

**Diptera:** In the order Diptera, an important family desired for population control is Culicidae (Suborder Nematocera), a clade representative of the main vector species of arboviruses and parasitic diseases in endemic areas. Muscoid dipteras of Suborder Brachycera, likewise, are potential targets for management.

Other families of sanitary importance, such as Ceratopogonidae and Psychodidae, are, to a lesser extent, studied in the context of the use of *M. anisopliae* for their population control to reduce the transmission of particular etiological agents. Ceratopogonidae is a family whose medical and veterinary importance comes from the ability of many species of the genus *Culicoides* to transmit viruses (such as the cattle bluetongue virus: Reoviridae), nematodes of the family Filariidae, among other pathogens (Cazorla and Campos, 2020).

Psychodidae includes important sandfly species that are vectors of Leishmaniasis, the main ones being those of the genus *Lutzomyia* (New World) and *Phlebotomus* (Old World). The last advances in control of dipterans using EPFs are summarized in **Table 1**.

**Hemiptera:** The Hemiptera order contains numerous species of agricultural importance (cochineals, cicadas, aphids, cotton strainers), and vectors of pathogens (bed bugs and kissing bugs). Studies involving pathogen-host interactions, formulation development in laboratory, semi-field, field, and integrated management were performed for various families, as Pentatomidae, Delphacidae, Pyrrochoridae. Diaspididae, and Aphidae, among others.

Besides the research on hemipteran agricultural pests, the prospection of EPFs has also been conducted for the biological control of vectors, with focus on kissing bugs (Reduviidae: subfamily Triatominae) and bed bugs (Cimicidae).

Studies also consider the control of hemipteran pests and vectors, exploring the scope of application in different environments and physiological and molecular aspects of the pathogen-host interaction, providing more information regarding their management with mycopesticides (Fancelli et al., 2013; Negrete González et al., 2018; Moreno-Salazar et al., 2020, See **Table 1**).

**Acarina - Ixodida**: Another arthropod group of sanitary and economic relevance that is a target for populational management in agricultural and urban environments. Ticks are considered the ectoparasites of cattle and wild animals that cause significant morbidity in their hosts due to their vector capacity, responsible for transmitting pathogens such as protozoa, viruses, and bacteria, such as *Rickettsia* (Pérez de León et al., 2020). Among the most important tick families are Ixodidae (hard ticks) and Argasidae (soft ticks), encompassing key genera such as *Rhipicephalus*, *Amblioma*, *Ixodes, Ornithodorus*, and *Otobius* (Pérez de León et al., 2020).

The prospection of the alternative control of ticks using EPFs and their formulations has been performed in recent decades (see **Table 1**). The veterinary and medical relevance of ticks, as well as the problems inherent in chemical pesticides, require more detailed investigations and more profound questions for the future development of sustainable pesticides for employment in agriculture.

## Conclusions and perspectives

The increase in human populations entails risks, responsibilities, and changes on a global scale over many habitats. Due to the anthropic expansion, it is expected that by 2050, approximately 300 million hectares of land will be occupied for agricultural purposes (Schmitz et al., 2014). Besides the economic issues, zoonotic diseases have increased as a result of the expansion of human cities. Approximately 60% of emerging infectious diseases in endemic countries are zoonotic, contaminating men and animals, and about 72% of these diseases came from wildlife due to human invasion (Jones et al., 2008; Nyhus, 2016).

Aiming at optimal pest management in the agricultural environment, of vectors in the socio-sanitary scope, and the avoidance of the problems inherent to the use of chemical pesticides, the development of mycopesticides has been prospected since the end of the 19^th^ century, with the first description of an EPF. Since approximately 6% of mycoinsecticides are composed of fungi of the genera *Beauveria* and *Metarhizium* (species and variation *anisopliae*) (Mesquita et al., 2023).

Although EPFs are promising options for developing large-scale insecticides for crop pests and vectorial control, many innate limitations must be considered for the successful establishment of such products.

Difficulties for isolation and characterization of endophytic fungal species comprise a central problem that compromises advances in EPF prospection. Since many species and strains exhibit specific conditions for reproduction, growth, sporulation, and pathogenicity, identification and standardization of tests for efficacy screening may offer obstacles in a first moment. Fortunately, there are media-based, biochemical, and molecular methods capable of promoting a reliable characterization and specification for successive assays using *in vitro* and *in vivo* techniques (Tsui et al., 2011; Bamisile et al., 2021).

Natural abiotic conditions associated with geographical locations are also determinants for fungal pathogenicity, virulence, and dispersion potential. Climatic conditions, environmental fragmentation, soil type, mesophilic conditions, and solar incidence were shown to interfere with the physiology of fungal entomopathogens since many clades develop and disperse under average conditions of temperature, radiation, osmolarity, and humidity. According to reports, the probability of discovering new strains of fungal entomopathogens is greater in regions classified as remote and less affected by human activities, with few exceptions explored, in which the tropical conditions for EPF development might be preserved (Zimmermann, 2007; Dong et al., 2016; Bamisile et al., 2021).

Another restriction in implementing EPFs as microbiological agents and mycoinsecticides is the rapid decline of fungal efficacy during the temporal window which comprises recognition, infection and ends in colonization and sporulation. As mentioned, insects and other arthropod hosts can generate immunologic responses. Such physiological reactions, combined with harmful environmental conditions and the fungal specificity of action (as a generalist or specialist fungi), may minimize the control potential of EPFs over a determinate target organism in short periods, thus compromising its impact. Integrated approaches regarding the molecular characterization and genetic engineering of fungal strains, isolation of specialist EPF species, prospection of propagules delivery strategies using artifacts or synthetic products, and formulation with pesticides are viable methods for efficacy optimization of fungal control (Paula et al., 2018; Bamisile et al., 2021; Wang et al., 2021; Wakil et al., 2023).

Due to the low requirements for its effective growth and virulence maintenance, in addition to a simple reproductive cycle and heteroxenic capacity, *M. anisopliae* has been highly prospected as a candidate for biopesticides aimed at various pests and vectors. The genetic diversity of the species, reflected in the large number of strains and variations, also allows for more detailed investigations, at the molecular level, on pathogen-host interactions, which remain largely unknown.

As previously mentioned, due to new technologies of manipulation and genetic engineering (such as CRISPR Cas9 and electroporation insertions), it will be possible to improve these strains for the overexpression of factors of virulence, resistance to radiation, temperature, and desiccation. Additionally, exogenous genes may be inserted to express toxic proteins against various species of economic and medical importance (Fang et al., 2012; Zhao et al., 2016; Chen et al., 2017). This may make the application of EPFs available in various ways for different purposes, with significant environmental benefits.

Thus, further studies are expected over the next few years to elucidate the molecular mechanisms of EPFs and species of interest, to obtain alternative pesticides. For this, governmental and private investments in research are highly desirable, as well as collaborations between the most varied institutions of health, agriculture, and research on a global scale.
